# Modulation of Milk and Lipid Synthesis and Secretion in a3-Dimensional Mouse Mammary Epithelial Cell Culture Model: Effects of Palmitate and Orlistat

**DOI:** 10.3390/nu14234948

**Published:** 2022-11-22

**Authors:** Michael G. Ross, Ken Kobayashi, Guang Han, Mina Desai

**Affiliations:** 1 The Lundquist Institute at Harbor-UCLA Medical Center, 1124 West Carson Street, Torrance, CA 90502, USA; 2 Department of Obstetrics and Gynecology, David Geffen School of Medicine, University of California Los Angeles at Harbor-UCLA, Torrance, CA 90502, USA; 3 Department of Obstetrics and Gynecology, Charles R. Drew University, Los Angeles, CA 90059, USA; 4 Laboratory of Cell and Tissue Biology, Research Faculty of Agriculture, Hokkaido University, Sapporo 060-8589, Japan

**Keywords:** triglycerides, STAT5, fatty acid synthase, sterol regulatory element-binding proteins, lipoprotein lipase, 3-dimensional mammary epithelial cell culture model

## Abstract

Human milk synthesis is impacted by maternal diet, serum composition, and substrate uptake and synthesis by mammary epithelial cells (MECs). The milk of obese/high-fat-diet women has an increased fat content, which promote excess infant weight gain and the risk of childhood/adult obesity. Yet, the knowledge of milk synthesis regulation is limited, and there are no established approaches to modulate human milk composition. We established a 3-dimensional mouse MEC primary culture that recreates the milk production pathway and tested the effects of the major saturated fatty acid in human milk (palmitate) and a lipoprotein lipase inhibitor (orlistat) on triglyceride production. Positive immunostaining confirmed the presence of milk protein and intracellular lipid including milk globules in the cytoplasm and extracellular space. The treatment with palmitate activated “milk” production by MECs (β-casein) and the lipid pathway (as evident by increased protein and mRNA expression). Consistent with these cellular changes, there was increased secretion of milk protein and triglyceride in MEC “milk”. The treatment with orlistat suppressed milk triglyceride production. Palmitate increased milk and lipid synthesis, partly via lipoprotein lipase activation. These findings demonstrate the ability to examine MEC pathways of milk production via both protein and mRNA and to modulate select pathways regulating milk composition in MEC culture.

## 1. Introduction

Nearly 80% of women initiate breastfeeding their babies at birth, and over a quarter of infants are still in part breastfed at 12 months [1,2]. For the infant, breastfeeding reduces the risk of developing associated diseases (respiratory, gastrointestinal, and ear infections) as compared to formula feeding. The American Academy of Pediatrics and the WHO recognize exclusive human milk feeding as the “normative standards for infant feeding” for the first six months after birth [3].

Human milk is a highly variable and complex biological fluid that contains more than 200 identified components [4] within compartments of true solutions, colloids (casein micelles), membranes, membrane-bound globules, and live cells. Constituent categories include aqueous vs. lipid fractions and nutritive vs. nonnutritive constituents. On average, the human milk contains 3–5% of fat, 0.8–0.9% of protein, 6.9–7.2% of carbohydrates, and 0.2% of minerals. Human milk median caloric content is 66 kcal/100 mL [5,6] with an interquartile range of 62.0 to 72.5 kcal/100 mL, reflecting a significant individual variance. Triglycerides (TGs) make up 98% of milk lipid content and contribute 40–50% of human milk energy content [7]. The variability in milk fatty acid (FA) composition contributes to the variance in milk caloric content [8]. 

Human milk TGs result from three sources: endogenous fat stores (adipose tissue and liver), dietary lipids, and de novo synthesis by mammary epithelial cells (MECs) [7,9]. Milk long-chain FAs are derived from either diet or endogenous fat stores, as human MECs have limited ability to synthesize C18 FAs. These long-chain FA which primarily are carried by very low density lipoproteins are taken up by MECs following hydrolysis by lipoprotein lipase (LPL) [10]. MEC lipid secretion depends on the abundance of gene transcripts encoding key lipogenic regulatory factors including sterol response element binding protein 1 (SREBP1) and peroxisome proliferator-activated receptors (PPARγ). In contrast to long-chain FAs, MECs produce short- and medium-chain FAs (C6-C14) de novo with acetate (and a contribution of β-hydroxybutyrate) as the primary carbon source being absorbed through the MEC basolateral membrane [11]. Their synthesis is primarily catalyzed by fatty acid synthase (FAS) and acetyl CoA carboxylase, while acylthioesterase serves to terminate the elongation of FAs [12]. The lactogenic hormones (prolactin, insulin, cortisol) directly activate the JAK2–STAT5 signaling pathway [13,14], and activated STAT5 (pSTAT5) promotes milk synthesis [13].

As a result of the impact on serum lipids, both maternal BMI and diet are important determinants of milk FAs, lipid content, and total caloric content [5,6,15,16,17,18]. The BMI directly correlates with milk fat content [19]. Notably, elevated plasma levels of triglycerides [20], free FAs [21], and particularly, saturated long-chain FAs such as palmitic acid are evident in women with obesity during pregnancy [22]. Palmitate is the major saturated FA in human milk, representing 20–25% of FAs. In contrast to the BMI, the dietary effects are more complex. Whereas a maternal high-fat diet may increase total milk fat as a result of long-chain FAs, [23] a low-fat diet increases the de novo MEC synthesis production of short- and medium-chain FAs [24,25]. 

Despite mixed evidence that breastfeeding reduces the incidence of childhood obesity [26,27], among breastfed large-for-gestational-age infants, childhood obesity rates are nearly 50% greater than among formula-fed appropriate-for-gestational-age infants [28]. Even among exclusively breastfed appropriate-for-gestational-age infants, a subgroup develops early-onset obesity [29]. Among infants exposed to maternal diabetes in utero, those consuming the largest amount of human milk displayed the highest body weight at 2 years. Although the value of human milk is beyond question, the variation of milk composition in women with high BMI or high-fat diets may contribute to a rapid and excessive infant weight gain. Increased infant weight at 6 at 12 months of age is a significant predictor of childhood and adult obesity. We sought to investigate the mechanisms by which long-chain saturated FAs modulate milk lipid production.

We utilized a novel 3-D mammary epithelial cell (MEC) primary culture model to recreate the milk production pathway in vitro and examined the effects of exogenous palmitate (conjugated to albumin) on milk triglyceride content. We further determined the effects of exogenous palmitate on the protein and mRNA expression of key factors involved in milk lipid synthesis in MECs and secreted “milk” in the apical chamber. Lastly, we tested whether a lipase inhibitor could prevent palmitate-induced milk production in MEC culture. The key findings of palmitate-mediated increased MEC synthesis and production of “milk” triglycerides, and its prevention by a lipase inhibitor provide evidence that the 3-D mouse MEC primary culture can be used to study “milk” secretion in vitro. This has implications for modulating human milk production and composition by maternal diet or pharmacologic intervention for optimal infant nutrition and growth. Furthermore, the cellular MEC responses of mRNA activation evident in secreted “milk” suggest that there is a potential to monitor human mammary cell responses via milk mRNA content.

## 2. Materials and Methods

Animals: Studies were approved by the Animal Care and Use Committee of The Lundquist Institute at Harbor University of California, Los Angeles, and were in accordance with the American Association for Accreditation of Laboratory Care and National Institutes of Health guidelines. Virgin female ICR mice (10–12 weeks of age; N = 6), purchased from Charles River Laboratories (San Diego, CA, USA), were housed in a facility at 22 °C with regulated humidity and a controlled 12:12 h light/dark cycle. The mice had free access to standard laboratory chow (Lab-Diet 5001, Brentwood, MO, USA) and water.

Materials: RPM1-1640 medium, protease inhibitor cocktail, BODIPY™ 493/503 (D3922), AlexaFluor™ 488 phalloidin (R37110), pSTAT5 (701063) and BAC™ Protein Assay Kit were purchased from Thermo Fisher Scientific (Irwindale, CA, USA). Trypsin, dexamethasone (mammalian D2915), epidermal growth factor (human E9644), insulin (bovine I6634), prolactin (mouse SRP4688), palmitate (sodium salt), fluorometric assay (MAK266), orlistat (O4139) and fluorescein isothiocyanate-dextran 3000 were purchased from Sigma-Aldrich (St. Louis, MO, USA).

The RIPA solution was obtained from Cell Signaling (Beverly, MA, USA), collagenase lll from STEMCELL Technologies Inc (Cambridge, MA, USA), fetal bovine serum from Corning Life Sciences, 0.4 μm pore size cell culture inserts, from BD Biosciences (La Jolla, CA, USA) and triglyceride assay kit from BioVision (K622; Milpitas, CA, USA). The primary antibodies anti-FAS (SC-20140) and anti-SREBP (SC-8984) were from Santa Cruz Biotechnology, Inc (Dallas, TX), the anti-β-casein antibody from Abbexa (abx430060, Sugar Land, TX, USA), and the anti-adipophilin antibody from LSBio (LS-C146977-100, Seattle, WA, USA). 

The growth medium consisted of RPMI-1640 supplemented with 10% FBS, 10 µg/mL of insulin, 10 ng/mL of EGF, 100 U/mL of penicillin, and 100 µg/mL of streptomycin. The lactogenic medium consisted of growth medium plus 0.5 U/mL of prolactin and 1 µM dexamethasone. The palmitate–albumin conjugate stock solution contained a palmitate solution (2 mM/L in 50% ethanol) and FA-free BSA (2%) and was diluted in lactation medium to provide 50 and 100 µmol/L palmitate concentrations. Orlistat was dissolved in ethanol.

**Figure 1:**
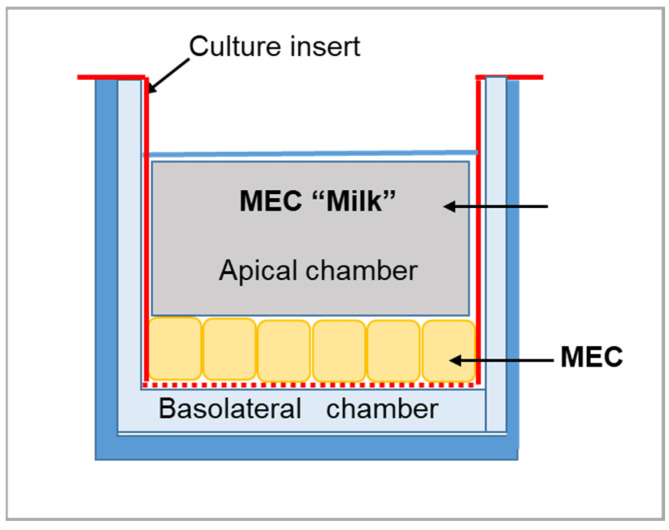
Schematic of the culture model. The cell culture inserts maintain the cell polarity of mammary epithelial cells (MECs), which secrete milk components into the apical (**upper**) compartment from nutrient sources in the basolateral (**lower**) compartment.

3-D Cell Culture Model: We used the method described by Kobayashi that recreates the milk production pathway in mammary epithelial cell (MEC) primary cultures [14,30,31]. The method uses cell culture inserts to maintain the cell polarity of MECs, which secrete milk components into the apical (upper) compartment from nutrient sources in the basolateral (lower) compartment (Figure 1). Briefly, the mice (N = 6) were euthanized, and the 4th abdominal mammary glands from both sides were collected in PBS buffer for the isolation of MECs. The mammary glands were minced and incubated in RPMI-1640 medium containing collagenase III (1.5 mg/mL) for 2 h at 37 °C with gentle shaking, followed by centrifugation (600× *g* for 1 min). The pellet was re-suspended in RPMI-1640 with 0.2% trypsin for 5 min at room temperature. The fragments of the mammary epithelium were dissociated by gentle pipetting with a Pasteur pipette and centrifuged (600× *g* for 1 min), and the pellet was re-suspended in 60% FBS (fetal bovine serum) followed by centrifugation (10× *g* for 1 min). The trypsin treatment and centrifugation with FBS were repeated for the isolation of mammary epithelial fragments without contaminating cells, such as fibroblasts, adipocytes, and myoepithelial cells. The epithelial fragments (0.1 mg) were subsequently seeded in 24-well cell culture plates with inserts to separate the basolateral chamber containing the lactogenic medium (nutrients) and the apical chamber containing the secreted MEC “milk”. The cells were cultured for 6 days at 37 °C in growth medium placed in the basolateral chamber. To induce lactogenesis, MECs were cultured in lactogenic medium placed in the basolateral and apical chambers for 2 days at 37 °C. To induce a high milk production ability, MECs were cultured at 39 °C with the lactogenic medium in the basolateral chamber and with RMPI-1640 in the apical chamber (Figure 1).

Treatments: Palmitate (50, 100 µmol/L) or the lipase inhibitor orlistat (100 µmol/L) together with palmitate (100 µmol/L) were added to the lactogenic medium within the basolateral chamber, and RPMI-1640 was maintained in the apical chamber. After 48 h, MECs and apical medium were collected separately for analysis, as described below. 

Sample Analysis: Henceforth, we refer to cellular MECs as “MECs” and to apical medium as MEC “milk”. MECs were fixed for immunostaining or stored at −80 °C for protein and mRNA expression studies. MEC “milk” was frozen at −80 °C and analyzed for triglyceride concentration and protein and mRNA expression.

Immunostaining: MECs were fixed with 1% formaldehyde in PBS at 4 °C and then immunostained for markers of epithelial cells (F-actin), milk (β-casein), lipids (BODIPY), milk lipid droplets (adipophilin), and nuclei (DAPI). PBS containing 5% bovine serum albumin was used to block nonspecific binding. Images of MECs were acquired using a fluorescence microscope. F-actin staining (AlexaFluor™ 488 phalloidin, R37110) was performed.

Western Blot: MEC protein was extracted, and protein expression was determined as previously described by us [32,33]. Briefly, the cells were harvested and dissolved in RIPA solution with a protease inhibitor cocktail and sonicated, and the cell lysates were processed for the analysis of protein concentration by the BAC™ Protein Assay Kit. For the detection of pSTAT5, the protein phosphatase inhibitor NaF (50 mM) was added to all buffers. MEC protein expression of β-casein (25 kDa), FAS (270 KDa), SREBP1 (68 KDa), and pSTAT5 (92 KDa) was analyzed.

MEC “milk” was similarly analyzed for protein expression of β-casein.

RT-PCR: The mRNA abundance of the target genes (lipoprotein lipase, LPL; FAS) and the reference gene (18S) in MEC and MEC “milk” was determined by RT-PCR as previously described by us [33]. Briefly, RNA was extracted following the manufacturer’s instructions, using the RNeasy Mini Kit (Qiagen, Valencia, CA, USA). Briefly, a guanidine-thiocyanate-containing lysis buffer and ethanol were added to the samples to promote the selective binding of RNA to the RNeasy membrane. The samples were then applied to the RNeasy Min spin column where RNA binding to the silica membrane enabled contaminants to be efficiently washed away. The purified RNA was then eluted in RNase-free water. The purity of RNA (A260/A280) for all samples was 1.8~2.0 as determined by spectrophotometry, indicating that the samples were pure and clean. PCR was performed in 96-well optical reaction plates (Applied Biosystems, Foster City, California) on cDNA equivalent to 0.5 µg of RNA in a volume of 20 μL, using Takara PrimeScript RT master mix (RR036A, TaKaRa Bio) at 37 °C for 15 min. The primer sequences used for RT-qPCR were: Lipoprotein lipase, LPL (NM_008509.2): Forward Primer (5’-3’)—GGACGGTAACGGGAATGTATG; Reverse Primer (5’-3’)—ACGTTGTCTAGGGGGTAGTTAAA. Fatty acid synthase, FAS (NM_007988): Forward

Primer—GATGGAAGGCTGGGCTCTA; Reverse Primer—GAAGCGTCTCGGGATCTCTG. 18S: Forward Primer—GGACAGGATTGACAGATTGATAGC; Reverse Primer: TGGTTATCGGAATTAACCAGACAA. 

PCR (TaKaRa TB Green Premix Ex Taq, RR420) was performed for the genes of interest and the 18S gene, using the ABI-Prism 7700 Sequence System (Applied Biosystems) at the following conditions: 30 s at 95 °C for 1 cycle and 5 s at 95 °C, 30 s at 60 °C for 40 cycles. All samples were run in triplicate. In the control PCR, we replaced cDNA with water obtaining a threshold level (CT) value of 40, indicating no detectable PCR product under these cycle conditions. The ABI Sequence Detection System 1.6 software (Applied Biosystems) was used to select a threshold level of fluorescence that was in the linear phase of the PCR product accumulation. 

The results from the RT-PCR assay were calculated as the difference between the CT for a specific mRNA gene and the CT for the reference 18S mRNA and expressed as fold change, using the formula 2^−(ΔΔCT)^.

Triglyceride: MEC “Milk” concentration was analyzed using a fluorometric assay (MAK266, Sigma, St. Louis, MO, USA).

Cell Permeability: To assess whether mammary epithelial permeability was altered by the treatments, we measured the unidirectional flux of fluorescein isothiocyanate (FITC, molecular weight 376) and FITC-labeled dextran 3000 (FITC-dex) from the basolateral to the apical compartments [14,34]. MECs were prepared as stated above and treated with palmitate and orlistat as described above. After 2 days, FITC and FITC-dex (0.5 mg/mL) were added to the basolateral chamber, and the cells were incubated for 1 h. Thereafter, the basolateral and apical media were aspirated separately, and fluorescence intensity was measured at 490/520 nm excitation/emission maxima. The permeability of the cells is expressed as percent of flux into the apical chamber/total FITC administered into the basolateral chamber. 

Statistics: Mammary glands were pooled from N = 6 mice for MEC cultures. Each treatment was performed in quadruplicate. NCSS statistical software was used for data analysis. The differences between treated and untreated MECs were compared using ANOVA with Dunnett’s post-hoc test.

## 3. Results

### 3.1. MEC Model and Synthesis of Milk

**Figure 2:**
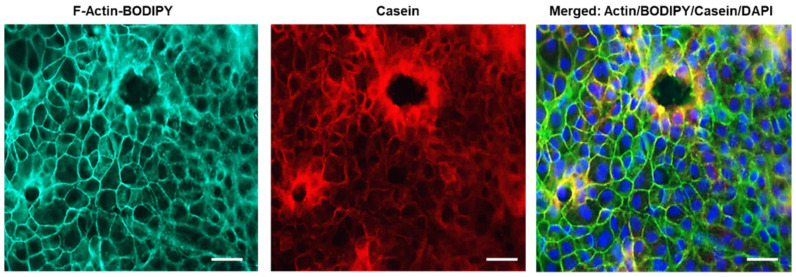
Milk Protein β-casein in Mice Mammary Epithelial Cultures (MEC). Immunostained images of F-actin (green) as a marker of epithelial cells and β-casein (red) as a marker of milk and merged image. Blue represents the nuclei stained with DAPI. Scale bars are 20 µm.

The culture model was evaluated by DAPI nuclear staining and F-actin, which confirmed the overall intact shape and structure of the mammary epithelial cells. The DAPI staining demonstrated intact MEC nuclei after lactogenic stimulation, while the staining of actin and β-casein (major milk-specific protein) showed a cytoplasmic localization of the latter, surrounding the nuclei, and confirmed the presence of milk as seen in the merged image (Figure 2 and Figures S1–S3). 

**Figure 3:**
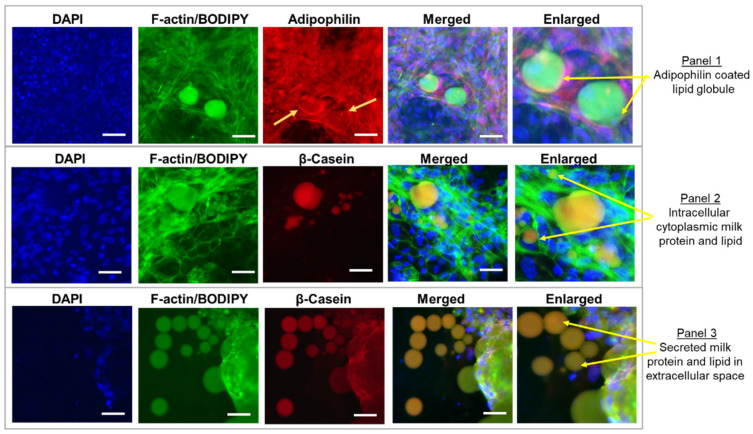
Milk Protein β-casein and Milk Lipid in Mammary Epithelial Cell (MEC) Cultures. Panel 1 shows immunostained MECs images for DAPI, F-actin/BODIPY, adipophilin, with merged and enlarged images. Panels 2 and 3 show immunostained MECs images for DAPI, F-actin/BODIPY, β-casein, with merged and enlarged images. Scale bar 10 µm.

BODIPY (a marker of neutral lipids such as triglycerides) demonstrated the presence of intracellular lipid, while co-staining with adipophilin (surface protein that coats the milk lipid globules) confirmed the presence of milk globules (Figure 3, Panel 1). Co-staining of the milk globules with casein confirmed the co-localization of milk protein and lipid in the globules and demonstrated their cytoplasmic localization (Figure 3, Panel 2). Additional staining of β-casein together with F-actin and BODIPY confirmed the secretion of milk protein and lipid in the extracellular space of in vitro-cultured MECs (Figure 3, Panel 3).

**Figure 4:**
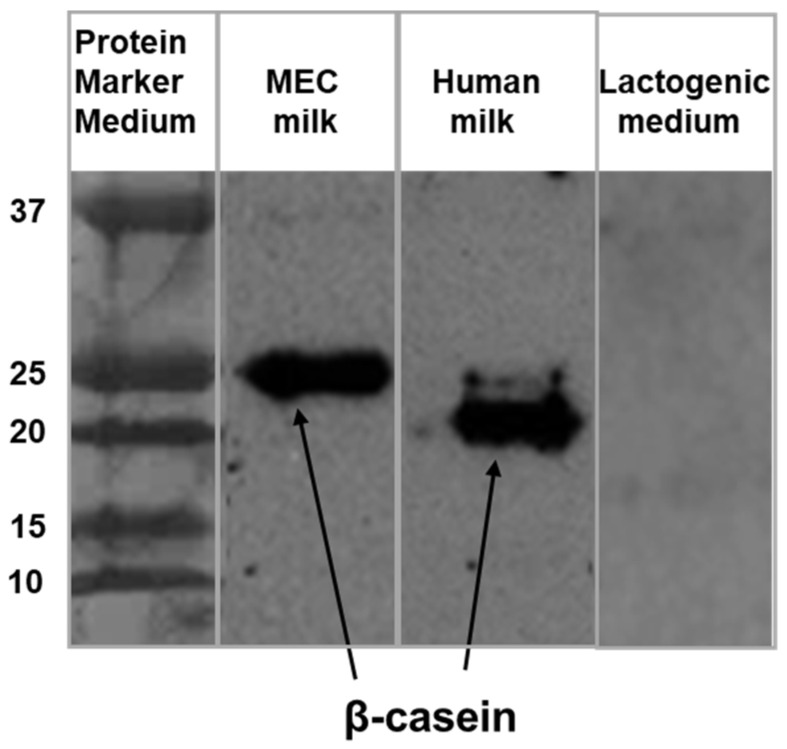
Secreted Milk Protein β-casein in the Apical Medium. Immunoblot of β-casein in MEC “milk” (mouse 25 kDa), human milk as a positive control (23 kDa), and lactogenic medium (no band detected).

The secretion of “milk” in the culture model was further verified using Western blot for the detection of β-casein in the apical medium. Consistent with the positive control (human milk β-casein, 23 kDa), mouse β-casein in the apical medium was detected at 25 kDa (Figure 4). No β-casein was detected in the lactogenic medium.

### 3.2. Provision of Palmitate Increased Milk and Lipid Synthesis

**Figure 5:**
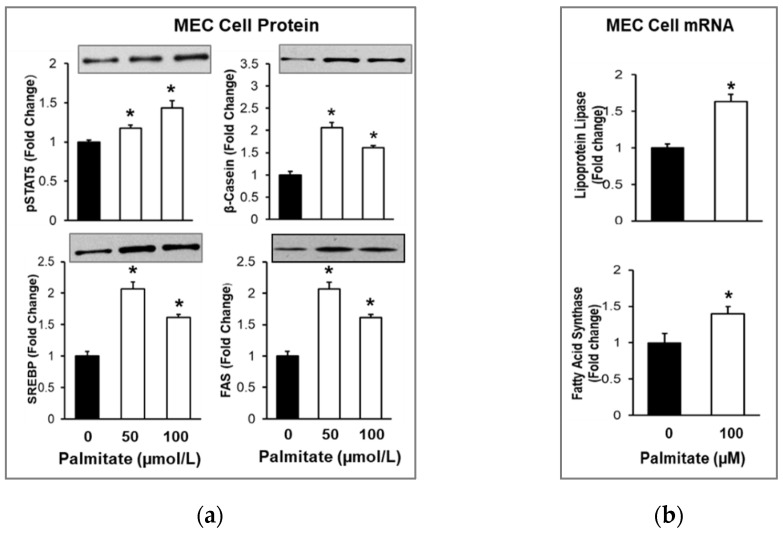
Effects of Palmitate on Milk Protein β-casein and Lipid Synthesis in Mammary Epithelial Cells. MECs were treated with varying doses of palmitic acid added to the lactation medium in the basolateral chamber for 48 h. Cellular MECs were analyzed for (**a**) protein expression (representative immunoblot shown) and (**b**) mRNA expression of milk lipid regulators. Each treatment was performed in quadruplicate, and differences between treated and untreated MECs were compared using ANOVA with Dunnett’s post-hoc test. Values are fold changes (Mean ± SE); * *p* < 0.001 treated vs. untreated MECs.

The effects of an exogenous palmitate–albumin conjugate on the protein and mRNA expression of key factors involved in milk lipid synthesis in MECs and secreted “milk” in the apical chamber were studied. Following the addition of the palmitate–albumin conjugate to the lactogenic medium, MECs demonstrated increased milk and FA synthesis, as evident by the increased protein expression of the lactogenic transcription factor pSTAT5, the milk protein β-casein, the lipogenic transcription factor SREBP1, and the lipogenic enzyme FAS. Consistent with the protein expression, the mRNA expression showed similar changes, with increased mRNA expression of the long-chain-fatty-acid-uptake enzymes Lpl and Fas (Figure 5). 

**Figure 6:**
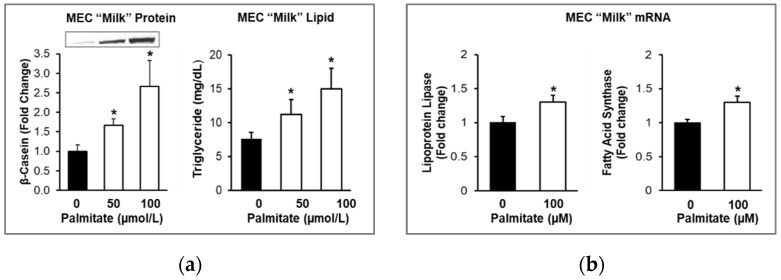
Effects of Palmitate on Secreted Milk Protein β-casein and Milk Lipid. MECs were treated with varying doses of palmitic acid added to the lactation medium in the basolateral chamber for 48 h. The apical medium was analyzed for (**a**) protein expression of β-casein (representative immunoblot shown) and triglyceride concentration. (**b**) mRNA expression of lipid enzymes. Each treatment was performed in quadruplicate, and differences between treated and untreated MECs were compared using ANOVA with Dunnett’s post-hoc test. Values are fold changes (Mean ± SE); * *p* < 0.001 treated vs. untreated MECs.

The analysis of MEC “milk” showed that with an increasing dose of palmitate, there was a dose-dependent increase in β-casein protein expression and triglyceride concentration. Notably, MEC “milk” mRNA expression of Lpl and Fas was also increased (Figure 6).

### 3.3. Treatment with Orlistat

**Figure 7:**
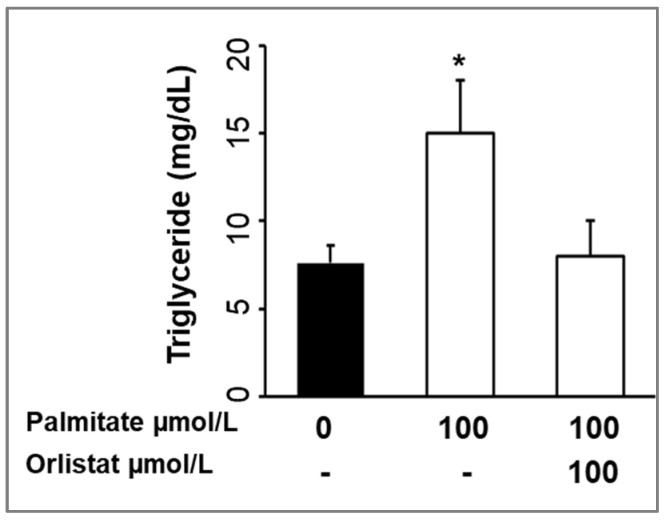
Effects of Orlistat on the Lipid Content. MECs were treated with palmitic acid and orlistat added to the lactation medium in the basolateral chamber for 48 h. MEC “milk” was analyzed for triglyceride levels. Each treatment was performed in quadruplicate, and differences between treated and untreated MECs were compared using ANOVA with Dunnett’s post-hoc test. Values are Mean ± SE; * *p* < 0.01 treated vs. untreated MECs.

We tested whether a lipase inhibitor prevented palmitate-induced milk production in the MEC culture. Orlistat treatment normalized the palmitate-induced MEC “milk” triglyceride concentrations to that of untreated MECs (Figure 7).

### 3.4. Treatments and Cell Permeability

**Figure 8:**
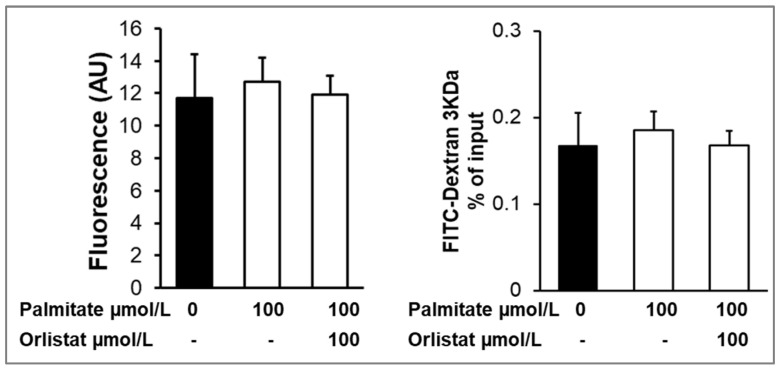
Treatment with FITC and FITC-Dextran. MECs were treated with palmitic acid and orlistat added to the lactation medium in the basolateral chamber for 48 h. FITC or FITC-dex was added to the basolateral chamber, and the cells were incubated for 1 h; the basolateral and apical media were aspirated separately, and fluorescence intensity was measured. Values are AU for FITC and percent of flux into the apical chamber/total FITC (Mean ± SE). Each treatment was performed in quadruplicate.

To evaluate the effect of the palmitate and orlistat treatments on epithelial barrier function, cell permeability was evaluated by measuring the unidirectional paracellular fluorescein flux and the leakage of FITC-dextran from the basolateral to the apical compartment. The fluorescence measured in the apical chamber was comparable between non-treated and treated groups. Additionally, the percent of flux of FITC-dextran from the basal to the apical chamber was overall minimal (<0.2%) and comparable between the groups, suggesting that the epithelial barrier function was not altered by the treatments (Figure 8).

## 4. Discussion

Numerous reports have confirmed that childhood obesity is a major risk factor for adult obesity [35,36] and that the children of overweight/obese (OW/OB) parents have an increased risk of obesity [37]. The underlying mechanism of programmed offspring obesity is a result of early developmentally increased appetite drive and reduced satiety with excessive weight gain during the nursing period [38,39]. Contributing further to infant OW/OB is the finding that the milk of women with OW/OB is high in fat and calories [15,19]. Animal studies confirmed that mice born to and nursed by obese/high-fat-diet dams demonstrated early-life and adult obesity. However, if the offspring of obese/high-fat-diet dams are cross-fostered and nursed by control (non-obese/normal diet) dams, they grow to a normal weight as adults. Remarkably, offspring of control dams who were cross-fostered and nursed by obese/high fat diet dams also demonstrated early-life and adult obesity [40,41]. These findings suggest that a compendium of factors (newborn growth trajectory, intrinsic infant appetite, breast milk nutrient, and caloric content) contribute to maintain the enhanced newborn growth [42]. Hence, knowledge of the mechanisms of milk synthesis/production and approaches to modulate milk composition is critical to prevent excess infant weight gain. The current study is an initial step to address this issue and used a 3-dimensional mouse MEC primary cultures to recreate the milk production pathway in vitro.

Consistent with the results of Kobayashi [30], our studies confirmed that the mouse MEC culture simulated the production of mammary gland milk production in vivo. Within epithelial cells, actin filaments are distributed predominantly at the plasma membrane and are highest in concentration at the apical membrane [43]. MEC immunostaining for F-actin confirmed the overall intact shape and structure of the mammary epithelial cells. Furthermore, the positive staining of β-casein confirmed the presence of milk proteins. MECs further demonstrated the presence of milk lipids and intact milk fat globules in both intracellular and extracellular compartments. Neutral lipids (stained by BODIPY) mainly in the form of triglycerides are synthesized and packaged into cytoplasmic lipid droplets [44] which are coated by the adipophilin protein [45]. In human and mouse milk, adipophilin co-localizes with secreted milk lipid globules and is significantly associated with the milk lipid content [45,46]. 

Consistent with MEC expression of β-casein, evidence of casein protein (25 kDa) in the apical-chamber milk was evidence of MEC “milk” secretory activity. Of note there was no evidence of casein in the lactogenic medium. 

Palmitate, the most common saturated FA accounting for 20–30% of total FAs in the human body, is obtained via the diet or synthesized endogenously via de novo lipogenesis [47]. Hence, its serum concentration in patients varies depending upon diet and physiological conditions [48], with higher levels demonstrated in patients with increased BMI [49]. The normal levels of adult serum triglycerides are less than 1.7 mmol/L (150 mg/dL) with normal nonesterified FA (free FA) levels approximating 10% of the triglyceride levels, of which palmitate comprises approximately 30% (~0.06 mmol/L). The lactogenic medium contained only 0.08 mmol/L of triglyceride and 0.03 mmol/L (0.84 mg/dL) of free FA, of which 21% was palmitate (0.006 mmol/L). The present study supplemented the lactogenic medium with palmitate FA at 0.05 and 0.1 mmol/L, reflecting the normal serum palmitate concentrations. We limited the palmitate concentrations to 0.1 mmol/L as levels greater than this may have lipotoxic effects in porcine MEC cultures [50,51]. 

The addition of palmitate to the lactogenic medium stimulated MEC “milk” production, as evidenced by the increased casein synthesis and secretion of both casein and triglyceride. The addition of Na-palmitate at 100 μmol/L with a molecular weight of 278 g (2.78 mg/dL) doubled the secretion of MEC triglyceride, increasing “milk” concentration by ~7 mg/dL. These findings are consistent with an in vivo study of dairy cows, as a short-term (3 weeks) and long-term (7 weeks) palmitate supplementation resulted in enhanced milk yield and increased milk fat content, with no effects evident on other milk components. Importantly, the increase in milk fat was exclusively due to an increased palmitate (C16) and palmitoleate (C16:1) incorporation into milk fat [52,53]. However, in the current study, the specific FA composition of the increased triglyceride remains to be elucidated. Further, whether OW/OB women have increased milk casein levels is not known, though a study on infant milk intake and body composition reported that increased human milk casein intake, and not total protein or whey intake, was related to lower infant lean mass and higher adiposity [54]. 

MEC triglyceride synthesis is dependent upon the uptake of FAs from the lactation medium. The uptake of long-chain FAs from albumin-bound FAs and lipoproteins is facilitated by a membrane lipase and FA transport proteins. The enzyme LPL functions to hydrolyze circulation-derived lipids into free FAs, which are subsequently transported as free FAs into cells by CD36 [9]. Consistent with evidence of a stimulatory effect of palmitate on both MEC uptake and synthetic pathways, palmitate (conjugated to albumin) supplementation increased the mRNA expression of MEC Lpl (long-chain FA uptake enzyme) and Fas and the protein expression of FAS, pSTAT5, and SREBP. Prolactin/STAT5 signaling plays a central role in milk production ability in MECs. Prolactin binds to the prolactin receptor in MECs, and STAT5 is then phosphorylated through Janus-Activating Kinase 2 (JAK2). Phosphorylated STAT5 forms dimers that translocate into the nuclei to regulate the transcription of genes related to milk production [55]. Palmitate-enriched diets cause SREBP1 activation [56], and SREBP1 facilitates triglyceride synthesis in MECs [57]. Palmitate may have additional effects on MEC activity, as it exerts multiple physiological functions at the cellular level. For example, palmitate stimulates skeletal muscle glucose uptake via AMPK and Akt activation [58], increases CCL4 expression in monocytes [59], and increases the secretion of pro-inflammatory cytokines [60]. Importantly, the increase in MEC cellular Lpl and Fas mRNA was reflected by increased mRNA in MEC “milk”. These findings are consistent with the studies of Haymond et al. [9] which demonstrated that milk mRNA reflects MEC cellular activation. Accordingly, the milk mRNA profile may potentially be utilized as a marker of mammary gland activation. 

The addition of orlistat, an LPL inhibitor, reversed the effects of palmitate stimulation on MEC “milk” triglyceride. Orlistat is used as an anti-obesity drug and acts by binding to the active sites of LPL, thus inhibiting the hydrolysis of triglycerides to free FAs. When used in humans [61], orlistat acts primarily within the gastrointestinal tract, as there is minimal systemic absorption [62]. Treatment of in vitro cultures at an orlistat-to-palmitate ratio of 1:1 [63] demonstrated cell viability using an orlistat concentration of 250 µM [64]. More recently, orlistat was also shown to be a potent inhibitor of FAS [65].

Palmitate stimulated both LPL and FAS, and treatment with orlistat suppressed MEC triglyceride production. We speculate that palmitate may have a stimulatory effect on LPL, with the increase in FAS representing a response to increased fatty acid substrates.

## 5. Conclusions

This study reported several key findings. Firstly, cellular MEC responses of mRNA activation were evidenced in secreted milk, indicating the potential to monitor human mammary cell responses via milk mRNA content. Secondly, palmitate activated MEC cellular properties, including uptake (LPL) and synthetic (FAS) pathways, rather than solely providing a substrate for triglyceride production. Finally, orlistat suppressed milk triglyceride production, and we speculate that the LPL pathway may be critical for the mechanism of action of palmitate. 

In view of the increased concentration of FAs in women with increased BMI or in response to a high-fat diet, it is likely that both diet and BMI are in part responsible for the increased fat content and caloric content of breast milk in these individuals. As increased human milk fat and caloric content may contribute to excess infant weight gain, it may be of value to reduce breast milk fat content. The use of select agents targeting maternal serum composition and MEC uptake, as well as synthesis and secretory pathways may be of benefit in modulating breast milk composition and preventing overweight/obesity in select newborns. As such, the current study provides preliminary evidence for a mouse cell culture system that could be used to study the regulation of lactogenesis and the modulation of milk composition. It provides a basis for establishing and testing diverse nutrients or pharmacological agents in MEC cultures to obtain milk with optimal nutrition and growth properties.

## 6. Future Perspectives

The importance of human milk for infant growth and development is critical. Milk contains a diverse range of nutrients including immune factors, hormones, microbes, and metabolites which can vary in response to maternal BMI, the metabolic/physiologic milieu, and diet. As stated above, mothers with OW/OB have an increased milk fatty acid content, which contributes to increased milk caloric content, excess infant weight gain, and increased risk of childhood and adult obesity. Notably, the infants of diabetic mothers may also experience the onset of overweight and the subsequent development of type 2 diabetes [66,67]. In contrast, preterm infants may receive insufficient protein at early ages when additional protein is required for growth [68]. Despite the importance of human milk composition and its potential implications for early infant growth and developmental programming effects, knowledge of the mechanisms of milk synthesis/production remains markedly limited. Our established novel 3-D mouse MEC culture model recreates the milk production pathway in vitro and allows a unified analysis of MEC cellular responses, “milk” production, and “milk” secreted mRNA. The findings of the palmitate-mediated increased MEC synthesis and production of “milk” triglycerides and its prevention by a lipase inhibitor provide evidence that the 3-D mouse MEC primary culture can be used to modulate milk production/composition in vitro. Nonetheless, due to differences in milk production/composition in mice and humans [69,70,71], it is of utmost importance to establish similar 3-D human MEC cultures to investigate the mechanisms of human milk production and the contribution of serum substrates and MEC enzymatic pathways to milk FAs, protein, as well as lactose and oligosaccharides. An understanding of the mechanisms of mammary cell substrate uptake, milk synthesis, and milk secretion which result in the marked variability of human milk composition will lead to the development of mechanism-based therapies that may include pharmacologic interventions, to optimize human milk composition. This would be a critical first step in the development of personalized infant nutrition with the aim of reducing the calorie content and increasing protein, that would be beneficial for obese and preterm infants, respectively.

## Supplementary Materials

The following are available online at https://www.mdpi.com/article/10.3390/nu14234948/s1, Figure S1: Images of Mice Mammary Gland Epithelial Cultures Prior and After Differentiation; Figure S2: Confirmatory Immunostaining Images of Mice Mammary Gland Epithelial Cultures; Figure S3: Confirmatory Immunostaining Image of MEC Milk Lipid Globule.
