# Supplementary material for: Modulation of Milk and Lipid Synthesis and Secretion in a3-Dimensional Mouse Mammary Epithelial Cell Culture Model: Effects of Palmitate and Orlistat
Source: Nutrients. 2022 Nov 22;14(23):4948. doi: 10.3390/nu14234948 (PMC9739267; doi:10.3390/nu14234948)
Supplement: Supplementary file 1 [file nutrients-14-04948-s001.zip › nutrients-1983052-supplementary.pptx]

## Slide 1
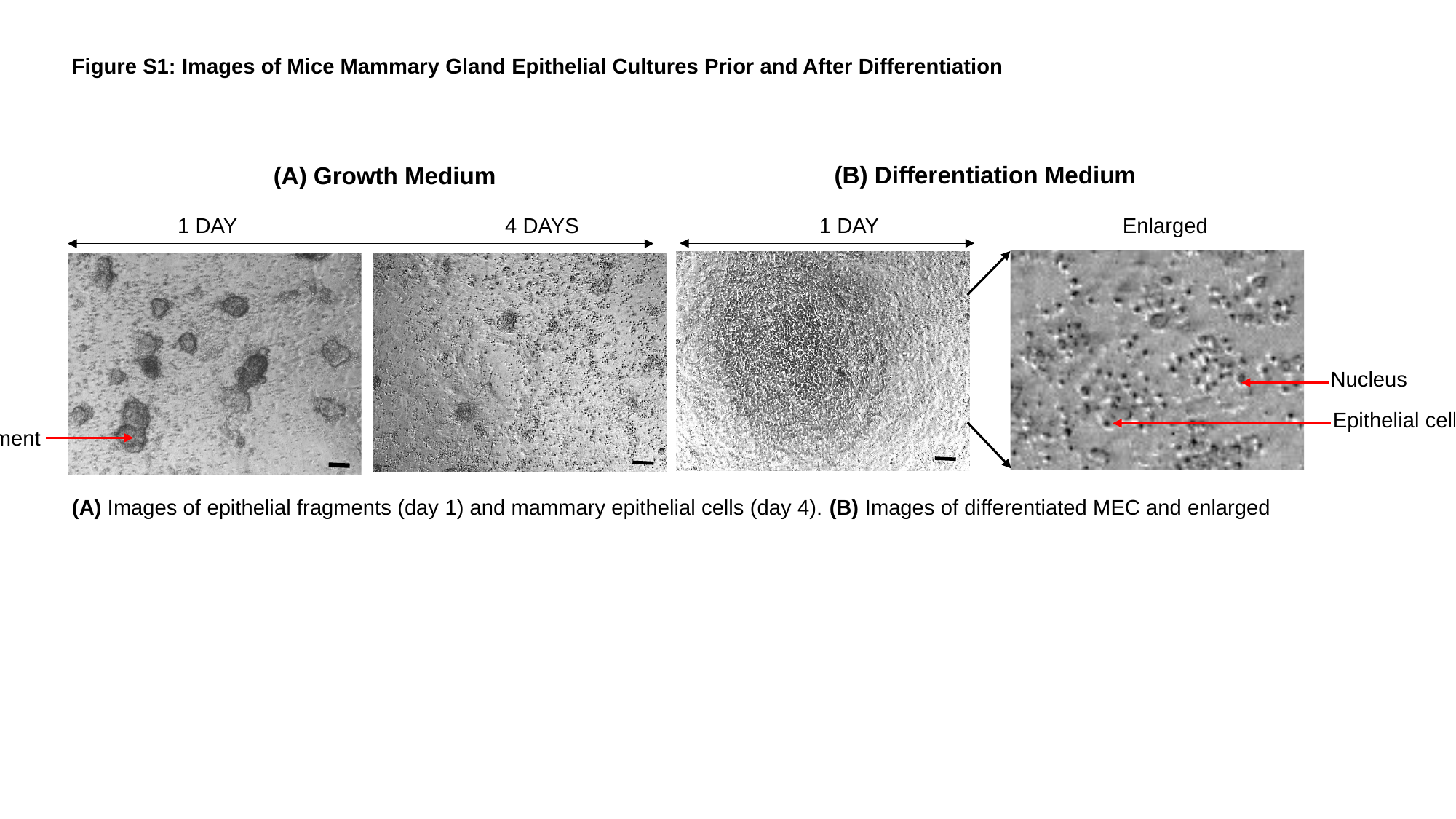

Figure S1: Images of Mice Mammary Gland Epithelial Cultures Prior and After Differentiation
(B) Differentiation Medium
(A) Growth Medium
1 DAY			4 DAYS		 1 DAY		 Enlarged
Nucleus
Epithelial cell
Fragment
(A) Images of epithelial fragments (day 1) and mammary epithelial cells (day 4). (B) Images of differentiated MEC and enlarged

## Slide 2
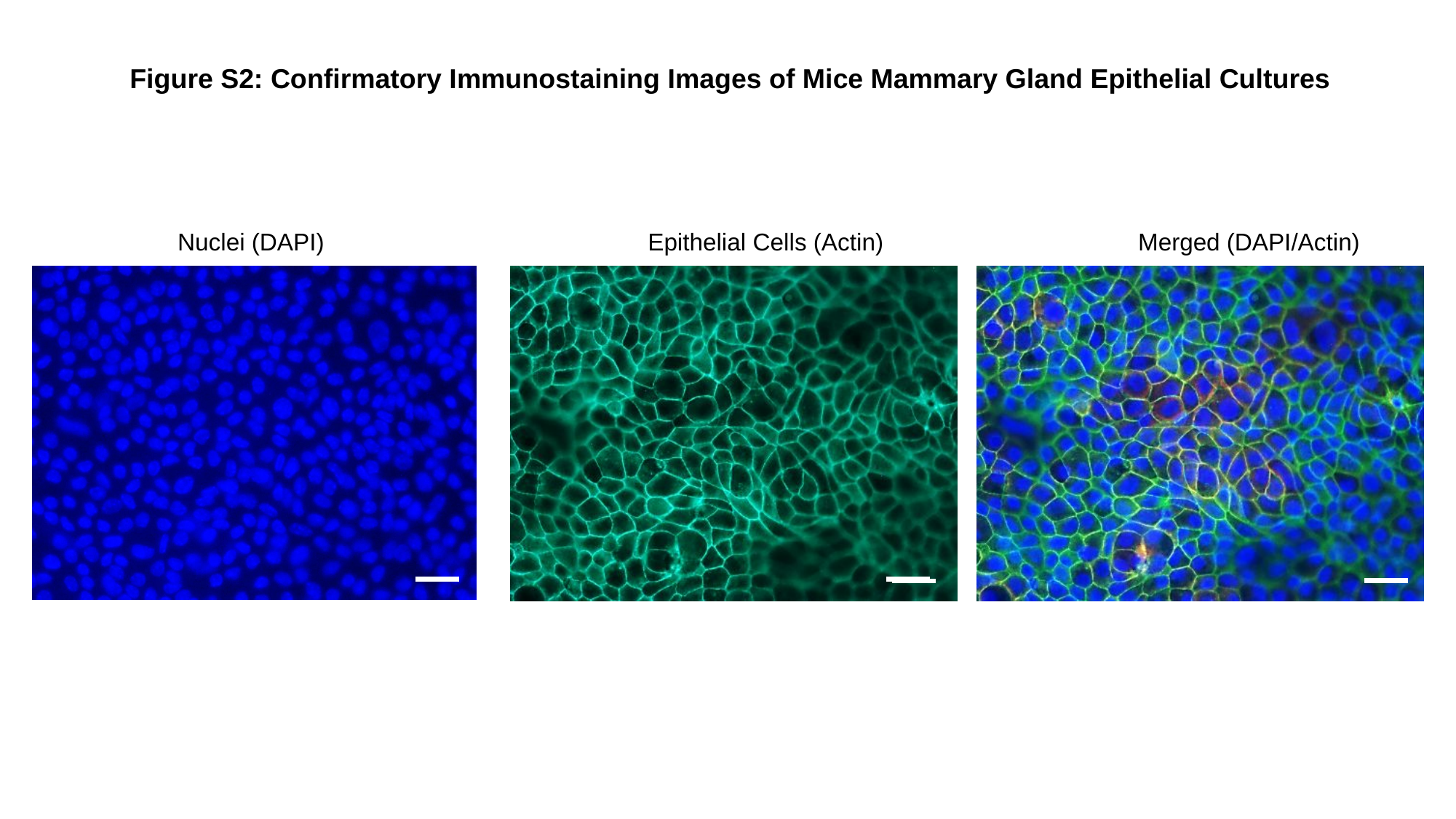

Figure S2: Confirmatory Immunostaining Images of Mice Mammary Gland Epithelial Cultures
Nuclei (DAPI) 			 Epithelial Cells (Actin)		 Merged (DAPI/Actin)

## Slide 3
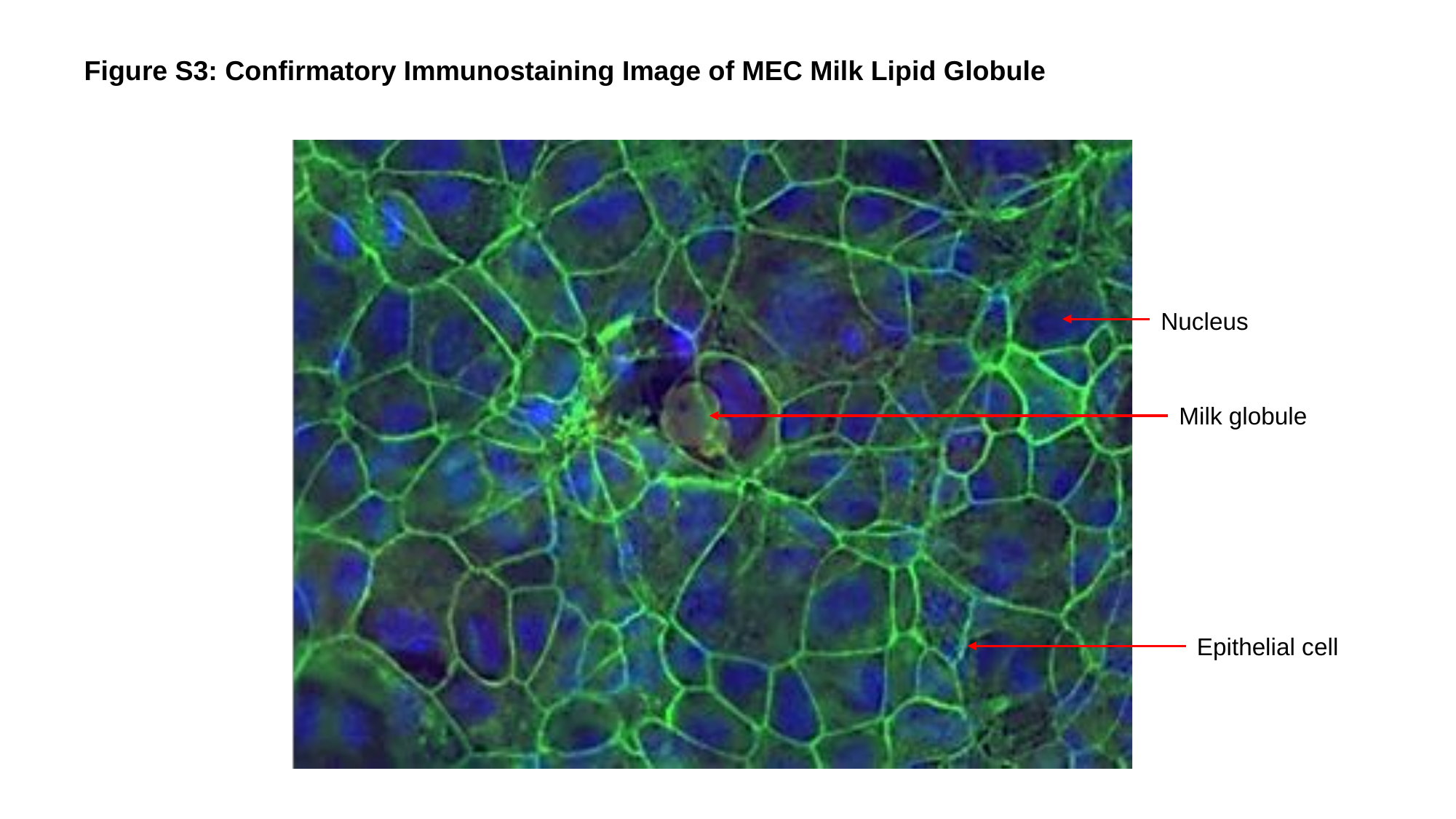

Figure S3: Confirmatory Immunostaining Image of MEC Milk Lipid Globule
Nucleus
Milk globule
Epithelial cell
